# The hypoxic microenvironment: a driving force for heterotopic ossification progression

**DOI:** 10.1186/s12964-020-0509-1

**Published:** 2020-02-07

**Authors:** Yifei Huang, Xinyi Wang, Hui Lin

**Affiliations:** 1 First Clinical Medical School, Nanchang University, Nanchang, 330006 Jiangxi Province China; 2 Department of Pathophysiology, School of Basic Medical Sciences, Nanchang University, 461 BaYi Avenue, Nanchang, 330006 Jiangxi Province China

**Keywords:** Heterotopic ossification, Hypoxic microenvironment, Hypoxia-inducible factor-1α

## Abstract

Heterotopic ossification (HO) refers to the formation of bone tissue outside the normal skeletal system. According to its pathogenesis, HO is divided into hereditary HO and acquired HO. There currently lack effective approaches for HO prevention or treatment. A deep understanding of its pathogenesis will provide promising strategies to prevent and treat HO. Studies have shown that the hypoxia-adaptive microenvironment generated after trauma is a potent stimulus of HO. The hypoxic microenvironment enhances the stability of hypoxia-inducible factor-1α (HIF-1α), which regulates a complex network including bone morphogenetic proteins (BMPs), vascular endothelial growth factor (VEGF), and neuropilin-1 (NRP-1), which are implicated in the formation of ectopic bone. In this review, we summarize the current understanding of the triggering role and underlying molecular mechanisms of the hypoxic microenvironment in the initiation and progression of HO, focusing mainly on HIF-1 and it's influenced genes  BMP, VEGF, and NRP-1. A better understanding of the role of hypoxia in HO unveils novel therapeutic targets for HO that reduce the local hypoxic microenvironment and inhibit HIF-1α activity.

Video Abstract. (MP4 52403 kb)

Video Abstract. (MP4 52403 kb)

## Background

Heterotopic ossification (HO) refers to the presence of bone tissue in muscle or connective tissue. When HO occurs in the joints, it causes swelling, pain, nerve compression and joint movement disorders. When HO occurs around the spine, it leads to limited spinal activity and spinal cord compression [1]. HO is a common complication after orthopedic surgery in patients with severe trauma [2, 3]. The pathogenesis of HO is not fully understood, but studies have proposed three necessary factors for HO formation: osteogenic precursor cells, multiple inducing factors and related signaling pathways, and an appropriate microenvironment [4, 5]. In particular, it should be noted that the initiation of HO requires appropriate local hypoxia in the microenvironment [6, 7].

Tissue damage and hypoxia are usually two simultaneous and related pathological conditions [8]. Hypoxia induces reactions including changes in organ structure, cytokine expression, and the immune response [9]. Studies have demonstrated that the reactions induced by hypoxia are involved in many diseases, such as cancer and HO [10, 11]. Hypoxia triggers the formation of HO, in which hypoxia-inducible factor-1α (HIF-1α) plays a crucial role [12, 13]. The most prominent feature under hypoxic conditions is high HIF-1α activity [14]. Upregulated HIF-1α modulates the expression of multiple genes, including bone morphogenetic proteins (BMPs), vascular endothelial growth factor (VEGF), and neuropilin-1(NRP-1), which in turn regulate biological processes such as angiogenesis, osteogenesis and bone resorption to induce ectopic bone formation [13, 15, 16]. This review will detail the trigger role and underlying molecular mechanisms of hypoxia in HO. Furthermore, we will highlight a powerful and potential therapeutic target for HO.

### Pathogenesis of heterotopic ossification

HO is divided into acquired HO and hereditary HO depending upon how HO forms and what causes it (Table 1) [3]. Acquired HO (aHO), which is usually induced by musculoskeletal trauma (such as burns, muscle damage and major joint surgery) and neurogenic trauma, can develop through mixed intramembranous and endochondral ossification [17, 18]. Musculoskeletal trauma-induced HO often occurs in soft tissues surrounding the injury, while HO caused by neurogenic trauma occurs away from the injury lesion [3, 19, 20]. There are two types of hereditary HO: fibrodysplasia ossificans progressiva (FOP) and progressive osseous heteroplasia (POH) [5]; FOP is due to a heterozygous mutation in the ACVR1 gene and involves endochondral ossification to form ectopic bone [21], while POH is caused by loss of function of the GNAS gene and involves ectopic bone formation through intramembranous ossification [3]. Adaptive microenvironment hypoxia occurs in soft tissue in response to severe trauma and initiates HO formation [12, 13]. A large number of inflammatory cells are recruited at the injury site, and ectopic ossification-related cytokines are released, these promote chondrocytes proliferation, cartilage extracellular matrix mineralization and osteoblast differentiation, then cartilage tissue is eventually replaced by ectopic bone [22, 23].

**Table 1:** The forms of heterotopic ossification

Classification		obtained form	Cause	Process of formation	
Inherited HO	FOP	Gene mutations	Gain of function of the ACVR1 gene	Endochondral ossification	
	PHO		Loss of function of the GNAS gene	Intramembranous ossification	
Acquired HO (AHO)		Injury	Injury to nervous system	Distal to injury	endochondral and intramembranous ossification
			Injury to musculoskeletal	Near the injury	

Heterotopic ossification (HO) can be divided into acquired HO and hereditary HO. Acquired HO usually results from damage to the nervous and skeletal muscle systems, while hereditary HO comprises FOP and POH, which is caused by genetic mutations in the ACVR1 gene or loss of function of the GANS gene. Acquired HO is developed by mixed intramembranous ossification and endochondral ossification. Ectopic bone formed near the injury site is induced by skeletal muscle injury, while ectopic bone formation away from the injury site is induced by nervous system injury. Hereditary HO develops through intramembranous ossification or endochondral ossification.

### Hypoxia and heterotopic ossification

Oxygen, an essential source of energy for cellular metabolism and maintenance of the body’s biological activities, is transported by the blood throughout body [24]. Hypoxia is the physiological and pathological process of tissue sedation, along with changes in cell metabolism, function and morphology due to insufficient oxygen supply or impaired oxygen use. Hypoxia may involve the whole body or only local tissue [25, 26]. Physiological hypoxia exists in many tissues of the human body, and the hypoxic environment is required for maintaining normal physiological functions, such as liver and bone [27, 28]. The hypoxic microenvironment is caused mainly by systolic vascular clogging due to inflammatory mediators release after severe trauma resulting in tissue blood circulation disorders and oxygen not being transported throughout the body [29]. In addition, the propagation of intracellular pathogens can cause hypoxia in infected cells [13].

**Fig. 1:**
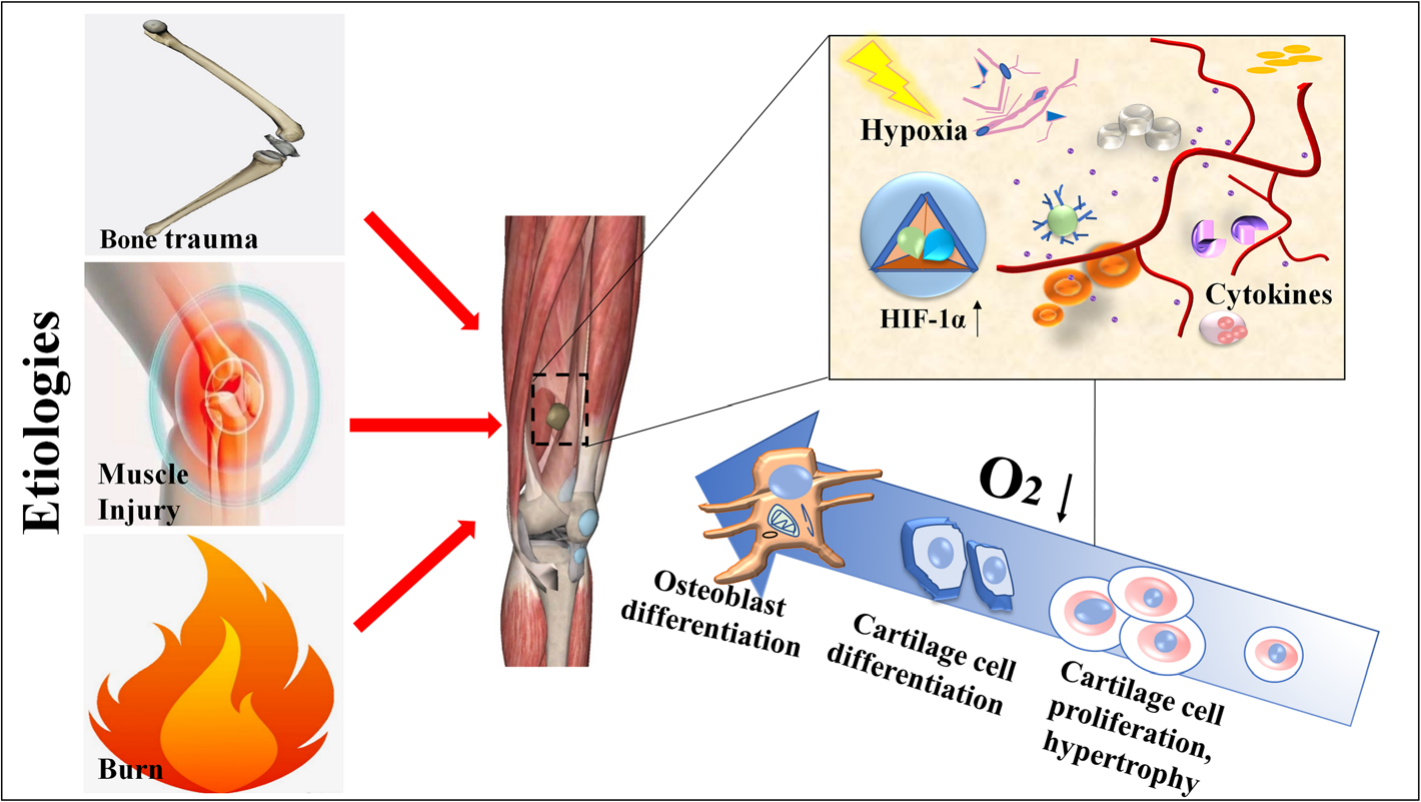
The role of hypoxia in heterotopic ossification (HO)

The effect of hypoxia is mediated by HIF-dependent and HIF-independent mechanisms [30, 31]. The HIF-independent pathway mainly causes alterations in protein phosphorylation status. As HIF is a transcription factor, the HIF-dependent pathway regulates downstream genes expression, thereby regulating biological processes, such as angiogenesis and apoptosis [32]. In the absence of oxygen, cell growth and the cell cycle are inhibited, and apoptosis is increased [33]. Moreover, the stability of HIF-1α protein is increased and the transcription is activated, which effectively induces target genes and enhances the body’s adaptation to hypoxic stress and environmental changes [32, 34]. Note that HIF-1α plays a crucial role in bone development and normal repair [35] Studies have shown that HIF-1α protein up-regulated by hypoxia not only maintains chondrocyte survival, promotes chondrocyte proliferation [36], participates in chondrocytes differentiation [37], and enhances matrix accumulation in chondrocytes, but also increases VEGF-A protein to generate blood vessels in cartilage, completing normal intrachondral osteogenesis [38]. However, under certain special circumstances, abnormal gene expression due to hypoxia has the opposite effect and creates the right conditions for various diseases, including our focus HO, metabolic disorders, inflammatory diseases and cancers [39–42]. Studies have indicated that HIF-1α with high stability plays an important role in the formation of heterotopic bone in cartilage, which promotes chondrocyte proliferation, hypertrophy and finally differentiate into osteocytes by regulating the expression of BMP, VEGF and other genes [22]. Hypoxia, which is accompanied by soft tissue trauma, is a driving force for the acquisition of acquired or hereditary HO [10, 43]. In the local hypoxic microenvironment, HIF is activated, which affects the expression of genes such as BMP, VEGF, and NRP-1 and promotes mesenchymal cell differentiation and ectopic bone formation [44, 45]. HIF activation is the trigger for hypoxia-induced heterotopic ossification (Fig. 1). Acquired HO is caused mostly by tissue damage via hypoxia -induction [7]. Vascular system damage, which leads to immune cell infiltration and proliferation and hence decreased oxygen supply and/or increased oxygen consumption, is often accompanied by tissue hypoxia [46]. The immune response, vascular endothelium, cartilage and bone tissue hyperplasia are all developed under hypoxic conditions [13]. Early ectopic ossification is accompanied by severe tissue hypoxia. In aHO patients, a large number of immune cells and HO precursor cells are present in lesions [47]. Furthermore, the expression of BMP, HIF-1α, VEGF and other cytokines involved in ectopic bone formation is upregulated [7, 12, 48]. Hypoxia is the main cause of this cellular recruitment and cytokine activation. In addition, in the inflammatory reaction after trauma, bacterial lipopolysaccharide inhibits proline hydroxylase and mediates the transcriptional activation of HIF-1α [49], further enhancing the expression of genes associated with ectopic bone formation, thereby aggravating induced HO [50].

Trauma, such as fractures, muscle damage, and burns, is an important cause of the occurrence of HO. When tissue is severely damaged, a local hypoxic microenvironment forms. Under hypoxic stimulation, HIF is activated and regulates the expression of cytokines, promoting the differentiation of ectopic bone precursor cells and mesenchymal cells, into cartilage and osteogenic cells, which ultimately develop into ectopic bone.

Hypoxia is also involved in the development of FOP [13]. Patients with FOP continue to develop ectopic bone after minor trauma [51], similar to HO induced by nonhereditary trauma. The early stages of ectopic bone formation are associated with active inflammation, immune responses, and the secretion of numerous cytokines [5]. Tissue hypoxia triggers the progression of HO in FOP [29]. One study found that FOP could directly upregulate BMP expression in connective tissue progenitor cells (CTPCs) independent of Activin A (Act A) in the hypoxic microenvironment to promote ectopic bone formation [52].

### Molecular mechanism of hypoxia-induced heterotopic ossification

**Fig. 2:**
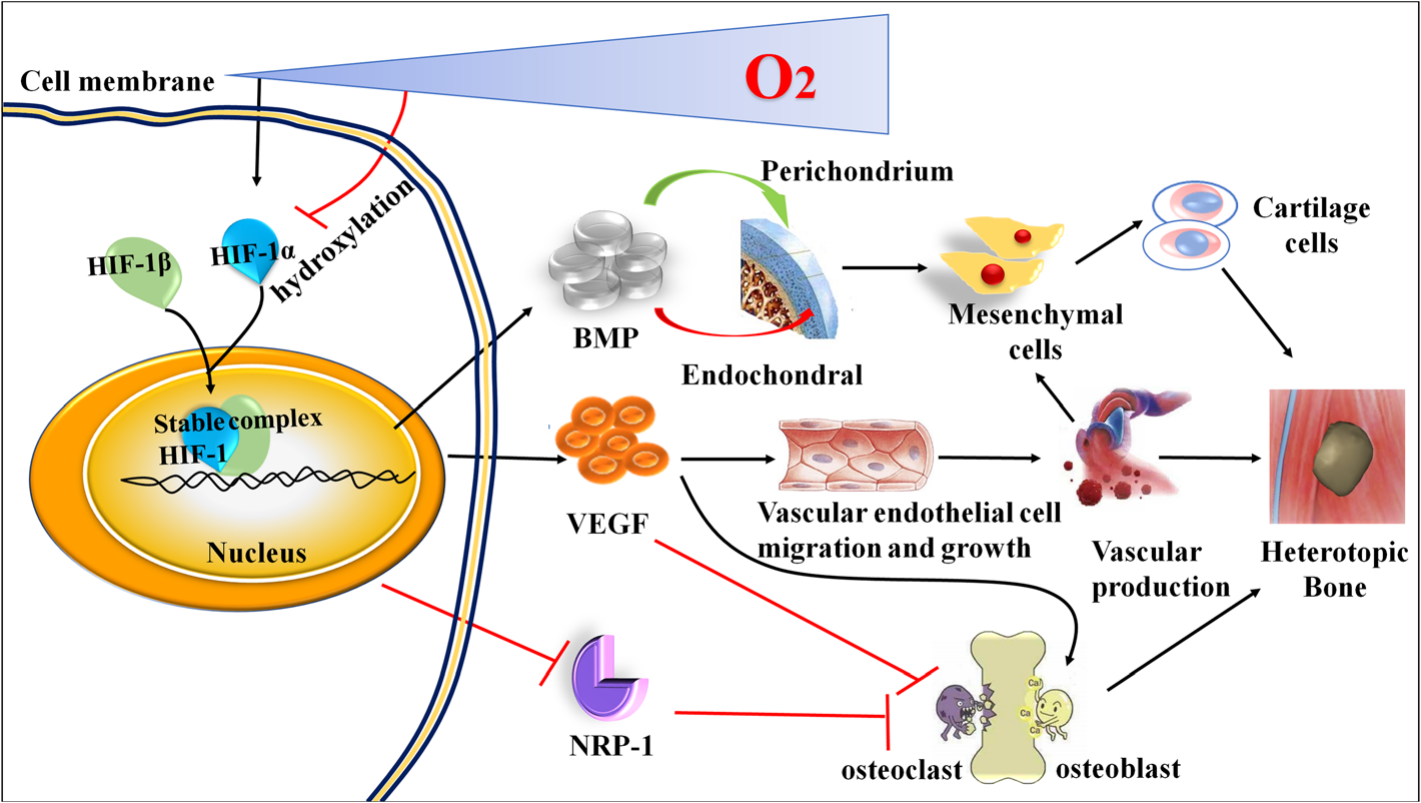
The specific molecular mechanisms of hypoxia-induced heterotopic ossification (HO)

The most direct effect of hypoxia-induced HO is the activation of HIF-1α, which regulates the expression of BMP, VEGF, and Neuropilin-1, thereby inducing angiogenesis, cartilage differentiation, and ultimately ectopic bone formation (Fig. 2).

HIF-1α is the core and hub of the whole process of HO. The hypoxic microenvironment mainly provides the necessary conditions for the stability of HIF-1α. The HIF-1α and HIF-1β complex up-regulates BMP and VEGF and down-regulates the expression of NRP-1, all these promote angiogenesis and the proliferation and differentiation of cartilage and osteoblasts and inhibit the proliferation and differentiation of osteoclasts, thereby inducing HO formation.

### Role of the HIF-1α signaling pathway in heterotopic ossification

The adaptation of cells in response to hypoxia caused by physiological or pathological conditions is mediated by genes that regulate angiogenesis and glycolysis, a phenomenon that is partly faciliated by the HIF family [53]. There are three HIF-α family members in mammals. HIF-1α is a crucial transcription factor related to oxygen homeostasis in the body [49]. Its stability is increased under hypoxia than under normal conditions. For example, trauma-induced HO usually produces necrotic tissue. Studies have found that necrotic tissue produced by injury has a lower oxygen level than healthy normal tissue [49, 54, 55]. HIF-2α is restricted to certain tissues [56], and HIF-3α is a negative regulator of HIF [57].

Under hypoxic conditions, HIF-1 α transcription is activated, which accelerates glycolysis and increases cell survival, inflammation, angiogenesis, and vascular permeability in tissues [58]. The stability of HIF-1α under hypoxic conditions has been found to recruit HO precursor cells, promote the proliferation and differentiation of mesenchymal stem cells and induce ectopic bone formation [7, 36]. Studies have shown that aHO and FOP proceed mainly through the HIF-1α-dependent BMP/SMAD signaling pathway or direct regulation of SOX-9 and Runx-2 expression [12]. In addition, VEGF is thought to amplify the BMP/SMAD signaling pathways to induce ectopic bone formation [15, 22].

In conditions such as hypoxia, signaling factors in the body influence gene transcription and translation, thereby accomplishing certain specialized biological functions. The HIF-1α and HIF-1β complex has been found to control the translation of three proteins, BMP, VEGF and NRP-1 [59–61], these three proteins promote angiogenesis and the proliferation and differentiation of chondrocytes and osteoblasts, providing the conditions for ectopic bone generation (Fig. 2).

### HIF-1α and BMPs

BMPs are widespread in the bone matrix, where they play an important role in growth and developmental processes, such as osteogenesis and glycolipid metabolism [62]. BMP signaling is mediated by a heterotetramer formed by two type I receptors and two type II receptors on the cell surface. It recruits and phosphorylates two downstream effectors (R-Smad), which form a heterotrimer with a Co-Smad. This complex then accumulates in the nucleus, where it regulates the expression of different genes [63]. BMP signaling is also mediated by non-Smad signaling pathways, such as the P38 MAPK signaling pathway [64]. BMPs usually induce bone formation through endochondral ossification, where mesenchymal cells in connective tissue first differentiate into cartilage tissue and then calcify into bone [65]. Bone formation can also occur via intramembranous ossification, where mesenchymal cells in connective tissue are spatially oriented, aggregate, and differentiate to form cartilage and bone [62].

BMPs are directly related to the occurrence of HO. Animal studies have shown that HO is caused by the over-expression of BMPs, especially BMP-2 and BMP-9, in osteoblasts during tissue damage [66, 67]. Rittenberg et al have reported that the inhibition of BMPs reduced HO [66]. Another study has found that blockade of the BMP-9 receptor significantly inhibited ectopic bone formation in damaged muscle [67]. In one clinical study, high levels of BMP-9 expression has been detected in the muscle tissue of patients with traumatic HO [68]. After mild trauma in FOP patients, BMP-4 is also significantly unregulated [69]. Hypoxia in aHO directly upregulates the expression of BMP to some extent, but hypoxia-activated BMP expression has been found to depend on HIF-1α [59]. The inhibition of HIF-1α signaling blocks the upregulation of BMP ligands induced by hypoxia [70, 71]. In trauma-induced HO in FOP, hypoxia affects mainly the type I receptor ACVR1 (mutant). Studies have shown that hypoxia stimulates oxygen sensors, causing the upregulation of HIF-1α, followed by endocytosis and inhibition of the degradation of cell surface protein kinase receptors. These effects lead to the retention of more mutant ACVR1 (mACVR1) on the cell membrane, prolonging the activation of BMP and enhancing the effect of BMP [59, 72].

### HIF-1α and VEGF

VEGF is a vascular endothelial cell mitogen that binds vascular endothelial cell surface receptors and activates their corresponding pathways to regulate angiogenesis directly [73, 74]. The main biological functions of VEGF are to promote angiogenesis, increase vascular permeability, and maintain normal blood vessels and integrity [75]. VEGF displays functional diversity in tissues through its effects on different receptors and co-receptors. Moreover, studies have found that the expression of VEGF is tissue-specific and context-dependent [76]. Under normal physiological conditions, VEGF is highly expressed in tissues with a high metabolic rate and sufficient blood supply, such as kidney, embryonic tissues and tissues in wound repair [77]. In the absence of oxygen, VEGF secretion is also greatly increased, which promotes the growth and migration of vascular endothelial cells and regulates bone and cartilage formation [78]. Accumulation of HIF-1α accelerates the release of VEGF cytokines, thereby stimulating blood vessel growth and allowing the body to obtain more nutrients and oxygen during hypoxia [79, 80].

Animal studies have shown that the use of exogenous VEGF in mice enhanced the formation of trabecular bone, indicating that VEGF promotes ectopic ossification [81, 82]. In contrast, the number of capillaries at chondrocytes and the formation of ectopic bone is reduced after inhibition of the VEGF activity in mice [83]. Studies have also found that VEGF is involved in endochondral ossification, primarily through its regulation of skeletal muscle cells and the proliferation and differentiation of osteoblasts and osteoclasts. VEGF reduces the number of osteoclasts, enhances the role of osteoblasts and attenuates the role of osteoclasts, thereby directly promoting bone formation [83, 84]. Given the critical role of VEGF in bone formation [85], it is reasonable to assume that under hypoxic conditions, HIF activation and its promotion of ectopic bone formation are associated with the high expression of VEGF.

### HIF-1α and Neuropilin-1

NRP-1, a receptor on the surface of osteoclast precursor cells [86], binds specifically to semaphorin 3A (Sema3A) secreted by osteoblasts and inhibits the immunoreceptor tyrosine-based activation motif (ITAM) co-stimulatory signaling pathway, which in turn inhibits osteoclast differentiation [87]. NRP-1, which is also expressed in endothelial cells, vascular smooth muscle cells and tumor cells, mediates VEGF downstream signaling pathways [88]. The hypoxic microenvironment has been found to inhibit osteoclast differentiation, which is also blocked by the expression of NRP-1 through HIF-1α [89]. Osteoclasts, the only known cells to date with only bone resorption functions, play an important role in the maintenance of bone homeostasis [90]. Impaired osteoclast differentiation affects bone metabolism and bone resorption [91]. Under physiological conditions, bone marrow is initially in a hypoxic microenvironment. Fractures and inflammation lead to reduced blood flow or oxygen supply and a more obvious pathological hypoxic state [92], which aggravates inhibition of NRP-1 expression and reduces the proliferation and differentiation of osteoclasts [93]. Therefore, we suspect that the abnormally low expression of NRP-1 in the hypoxic environment is one of the important factors that promotes bone formation and ectopic bone formation.

### Prevention and treatment of heterotopic ossification

**Fig. 3:**
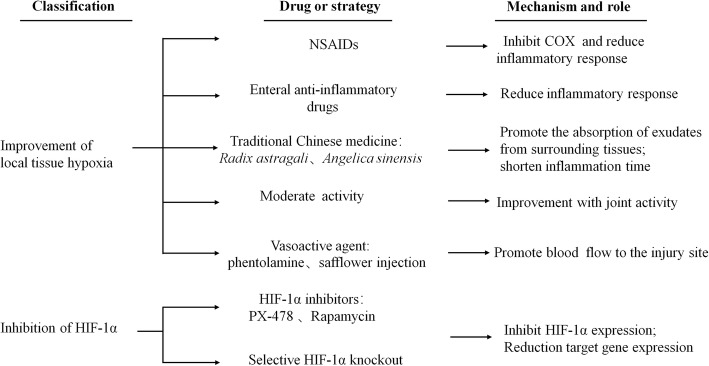
Strategies for the prevention and treatment of HO

In general, ectopic bone can be discovered only after it forms and is easily confused with bone tumors. Image-based examinations, such as bone scans, are generally used to make an accurate diagnosis [94]. Once HO occurs, the only effective treatment for HO is surgical resection and radiotherapy, but these treatments have many limitations, such as frequent HO recurrence, poor efficacy, and high cost [95]. Therefore, the principle of HO treatment should be prevention rather than treatment. Hypoxia is the key factor for the initiation of ectopic bone formation, and a promising strategy for HO could be management of the hypoxic microenvironment and inhibition of HIF-1α in the early stage of HO. Hypoxic action has allowed the following four treatment strategies for HO (Fig. 3).

Strategies for the prevention and treatment of HO are divided into two major categories: targeting of tissue hypoxia and direct inhibition of HIF-1α. Hypoxia is managed through increased blood circulation, anti-inflammatory drugs and moderate joint movements to improve blood circulation disorders and reverse the hypoxic microenvironment. HIF-1α is mainly down-regulated through HIF-1α inhibitors and the knockout of HIF-1α. We highlight the effects of PX-478 and rapamycin on the prevention and treatment of HO.

We systematically describe how hypoxia promotes ectopic ossification, and emerging mechanistic insights reveals promising strategies for the prevention and treatment of HO.

### Management of local hypoxia

Tissue hypoxia is inextricably linked to the posttraumatic inflammatory response and insufficient blood supply. The main reason for this link is that inflammation often releases inflammatory mediators that cause vascular blockage, blood circulation disorders, and the propagation of pathogens in infected cells after trauma induces hypoxia [13]. In addition, these inflammatory factors play important regulatory roles in the expression and activity of HIF-1α and its downstream targets [96–98]. Therefore, management of local hypoxia is mainly carried out through reducing inflammation and improving blood supply.

1. Nonsteroidal anti-inflammatory drugs (NSAIDs): NSAIDs have been used clinically for the prophylaxis of HO. The mechanism of action of NSAID is the delayed formation of HO by reducing the inflammatory response [99]. Commonly used NSAIDs include indomethacin and ibuprofen, but nonspecific NSAIDs are prone to developing peptic ulcers and other complications that have a greater adverse effect on the gastrointestinal tract. Hence so the use of selective COX-2 inhibitors is recommended for the prevention of HO [100].
2. Topical anti-inflammatory drugs: HO is induced after the patient experiences severe trauma. At present, the blood alkaline phosphatase concentration and erythrocyte sedimentation rates (ESR) are regularly tested in the clinic to determine the stage of ossification [101], especially when the ESR indexis high, which indicates that HO is in its active and late stages. The key to lowering the ESR index is to reduce inflammation of the wound with local ice application, as well as the external application of Qinghua Zhitong San and moderate debridement.
3. Oral and external use of traditional Chinese medicine: Oral and external use of traditional Chinese medicine improves microcirculation of the injured area and promotes the absorption of edema fluid, pooled blood and inflammatory exudate of the surrounding tissues [102]. Furthermore, the application of traditional Chinese medicine, such as *Angelica* and other antipyretic and analgesic Chinese herbal medicines relievethe degeneration of local muscle fibers and shorten the inflammatory reaction time [103].
4. Moderate activity: Clinical trials have shown that joint loosening during inactive ectopic ossification and complete maturation can effectively reduce ossification and help joint activity. Furthermore, in a rabbit model of trauma-induced HO, forced fixation of the animal’s body aggravated the occurrence of HO [104]. This finding indicates that after the soft tissue is traumatized and subjected to long-term compression, tissue hypoxia promotes HO due to poor blood circulation. Therefore, moderate activity is beneficial to prevent the occurrence of HO after injury.
5. Vasodilator drugs: Vasodilator drugs, such as phentolamine and the Chinese medicine safflower injection, increase the blood supply at the injured area and decrease the hypoxic microenvironment to prevent contracture and calcification of the joint capsule and surrounding muscles, preventing HO [105]. Clinical studies have shown that the use of safflower injection, like blood stasis drugs, has a certain inhibitory effect on traumatic HO [106].

### Inhibition of HIF-1α

PX-478 is a selective molecular HIF-1α inhibitor [107].PX-478 effectively inhibits the transcription and translation of HIF-1α under normal or hypoxic conditions [107], with translation inhibition as the main mechanism. PX-478 also inhibits HIF-1α deubiquitination. Rapamycin inhibits mainly the translation of the HIF-1α by blocking the mTOR signaling pathway, but does not alter the transcription of HIF-1α. Rapamycin is also very effective in inhibiting hypoxia-induced expression of mTOR and VEGF [108].

The study has shown that the inhibition of HIF-1α reduces HO precursor cells and decreases the expression of SOX-9, leading to a decrease in the volume of HO. No HO precursor cell aggregation or ectopic bone formation are observed after a conditional knockdown of HIF-1α in mice [12, 109]. Animal studies have found that the administration of PX-478 to an HO mouse model after 3 weeks significantly reduces cartilage protoplasts, as confirmed by histological evaluation. Furthermore, the study has found a clear decrease in the volume of ectopic bone when the fifth and ninth weeks are compared. PX-478 treatment completely inhibits HO of the soft tissue [12]. In addition, PX-478 down-regulates hypoxia-mediated VEGF expression(with no effect under normal conditions), but the effects of down-regulated VEGF expression under hypoxic conditions are apparent after approximately 8 h [110]. This property increases its potential for prevention and treatment of HO. Similarly, the use of rapamycin significantly reduces the production of trauma-induced and hereditary HO in mice, with some mice not showing any HO at all [12, 111]. After targeted knock out of HIF-1α in mice, the number of HO precursor cells is significantly reduced, and ectopic bone formation is reduced. Notably, rapamycin is now undergoing clinical trials for its use in HO [112].

## Conclusions

This review highlights the role of the hypoxic microenvironment in promoting HO, which primarily activates HIF and mediates members of its signaling pathways including BMP, VEGF, and NRP-1. First, hypoxia is a pathological process that emerges when tissue damage occurs; hypoxia and HIF-1α are inextricably linked. HIF-1α plays a central role in the regulation of multiple signal factors as follows. 1) The upregulation of BMP signaling factors promotes ectopic bone formation. 2) The upregulation of VEGF signaling promotes vascular endothelial growth and angiogenesis to regulate ectopic bone and cartilage formation. 3) The down-regulation of NRP-1 inhibits osteoclast differentiation and increases abnormal osteogenesis in bone metabolism. Therefore, it is reasonable to conclude that HIF-1α is a core hub in the signaling pathway that induces HO. Blockade of the HIF-1α activity would prevent the progression of HO. Since hypoxia is a direct cause of HIF-1α up-regulation, in addition to the use of HIF-1α targeting inhibitors, management of the hypoxic microenvironment could down-regulate HIF-1α and prevent HO. The hypoxic microenvironment is mainly related to inflammation and blood circulation disorders. Various drugs alleviate the inflammatory response and improve the ischemic state to change local hypoxia. These drugs can be used to prevent HO. Future methods to prevent and treat HO could be based on these findings:1) Tissue damage is a prerequisite of HO that promotes the progression of hereditary HO. Therefore, anti-inflammatory drugs and drugs that target diastolic blood vessels can be used to improve the hypoxic microenvironment at injured sites to prevent HO. 2) Exploration of the mechanism of HO should continue, and whether HIF-1α engages in crosstalk with other signaling pathways involved in HO should be investigated, which will strengthen HIF-1α as a targeted therapy for HO. 3) We should strive to stop HO in its early stages by improving hypoxia and inhibiting HIF-1α. Although the current understanding of hypoxia and HO remains very limited, we have nonetheless found that hypoxia and HIF-1α play a key role in the initiation of HO. This review describes explicitly how hypoxia induces HO and effective strategies against hypoxia and HIF-1α. Further understanding of the mechanism of HO will provide new strategies for HO treatment.

## Data Availability

Not applicable.

## Abbreviations

Act A: Activin A
AHO: Acquired HO Heterotopic ossification
BMPs: Bone morphogenetic proteins
COX-2: Cyclooxygenase-2
CTPCs: Connective tissue progenitor cells
FOP: Fibrodysplasia ossificans progressiva
HIF: Hypoxia-inducible factor
HIF-1α: Hypoxia-inducible factor-1α
HIF-1β: Hypoxia-inducible factor-1β
HIF-2α: Hypoxia-inducible factor-2α
HIF-3α: Hypoxia-inducible factor-3α
HO: Heterotopic ossification
ITAM: Immunoreceptor tyosine-based activation motif
mACVR1: Mutant Activin receptor A type1
mTOR: Mammalian target of rapamycin
NF-kB: Nuclear factor kappa beta
NRP-1: Neuropilin-1
NSAID: Nonsteroidal Antiinflammatory Drugs
POH: Progressive osseous heteroplasia.
Runx-2: Runt-related transcription factor 2
Sema3A: Semaphorin 3A
VEGF: Vascular endothelial growth factor
